# ISWI and CHD Chromatin Remodelers Bind Promoters but Act in Gene Bodies

**DOI:** 10.1371/journal.pgen.1003317

**Published:** 2013-02-28

**Authors:** Gabriel E. Zentner, Toshio Tsukiyama, Steven Henikoff

**Affiliations:** 1 Basic Sciences Division, Fred Hutchinson Cancer Research Center, Seattle, Washington, United States of America; 2 Howard Hughes Medical Institute, Fred Hutchinson Cancer Research Center, Seattle, Washington, United States of America; The University of North Carolina at Chapel Hill, United States of America

## Abstract

ATP-dependent nucleosome remodelers influence genetic processes by altering nucleosome occupancy, positioning, and composition. *In vitro*, *Saccharomyces cerevisiae* ISWI and CHD remodelers require ∼30–85 bp of extranucleosomal DNA to reposition nucleosomes, but linker DNA in *S. cerevisiae* averages <20 bp. To address this discrepancy between *in vitro* and *in vivo* observations, we have mapped the genomic distributions of the yeast Isw1, Isw2, and Chd1 remodelers at base-pair resolution on native chromatin. Although these remodelers act in gene bodies, we find that they are also highly enriched at nucleosome-depleted regions (NDRs), where they bind to extended regions of DNA adjacent to particular transcription factors. Surprisingly, catalytically inactive remodelers show similar binding patterns. We find that remodeler occupancy at NDRs and gene bodies is associated with nucleosome turnover and transcriptional elongation rate, suggesting that remodelers act on regions of transient nucleosome unwrapping or depletion within gene bodies subsequent to transcriptional elongation.

## Author Summary

Eukaryotic genomes are compacted into chromatin, which restricts access to DNA. In order for cells to transcribe, replicate, and repair DNA, chromatin structure must be altered. Eukaryotes have evolved chromatin remodeling enzymes that use energy derived from ATP hydrolysis to modulate chromatin structure. *In vitro*, yeast ISWI and CHD remodelers require 30–85 bp of extranucleosomal DNA in order to efficiently remodel chromatin, but *in vivo*, yeast linker DNA is, on average, <20 bp. By mapping yeast Isw1, Isw2, and Chd1 on native chromatin, we find that these remodelers bind to extended regions of linker DNA adjacent to transcription factor binding sites within nucleosome depleted regions. Remodeler binding is associated with nucleosome turnover and transcription rate, suggesting that ISWI and CHD remodelers help to reestablish proper chromatin structure following transcriptional elongation.

## Introduction

Nucleosome remodelers use the energy of ATP hydrolysis to alter histone-DNA contacts, slide nucleosomes, and exchange or remove histones and entire nucleosomes. They are ubiquitous throughout eukaryotic evolution [1], and alterations in their expression are found in human congenital anomaly syndromes and cancers [2]–[5], highlighting their central role in cellular life.

Yeast Isw1, Isw2, and Chd1 remodeler complexes bind ∼30–85 bp of extranucleosomal DNA, which is required for efficient nucleosome repositioning *in vitro* [6]–[10], and histone H3 depletion stimulates Isw2 nucleosome sliding *in vivo* [11], presumably by creating regions of accessible DNA. Additionally, the human ISWI remodeler SNF2h functions *in vitro* as a dimeric sensor of linker length, with one ATPase molecule contacting extranucleosomal DNA on each side of a nucleosome, and the efficiency of its nucleosome repositioning activity is decreased with decreasing linker length [12]–[14]. Similarly, the human CHD7 remodeler, mutated in the developmental disorder CHARGE syndrome [5], requires ≥40 bp of extranucleosomal DNA for remodeling *in vitro* [15]. Yeast Isw1, Isw2, and Chd1 act at various points in gene bodies [16]–[21], which contain arrays of regularly spaced nucleosomes [22]. However, the average nucleosomal repeat length in *S. cerevisiae* is 165 bp [22]. Given that 147 bp of DNA is wrapped around the histone octamer, only ∼18 bp of DNA is extranucleosomal. Thus, much more DNA on either side of a nucleosome is required for ISWI and CHD proteins to act than is available between nucleosomes. This paradox is even more severe in *S. pombe*, where the Chd1-like remodelers hrp1 and hrp3 act within gene bodies [23]–[25], but nucleosomal repeats average 154 bp and linkers are therefore only ∼7 bp [26].

To address this paradox, we mapped the genomic distributions of yeast Isw1, Isw2, and Chd1 on native chromatin. We show that yeast ISWI and CHD remodelers are highly enriched at nucleosome-depleted regions (NDRs) flanking transcription units, where they bind to extended stretches of linker DNA flanking transcription factor binding sites (TFBSs). We find that remodeler binding is positively associated with nucleosome turnover and transcriptional elongation rate, suggesting that ISWI and CHD remodelers first associate with naked DNA within NDRs and subsequently relocate to gene bodies following nucleosome disruption by RNA Polymerase II transit, upon which there is ample linker DNA to promote their efficient remodeling activity.

## Results/Discussion

### Genome-wide mapping of remodeler binding on native chromatin

We performed immunoprecipitation of uncrosslinked (native) chromatin digested with micrococcal nuclease (MNase) combined with high-throughput paired-end sequencing (N-ChIP-seq) for *S. cerevisiae* Isw1, Isw2, and Chd1. N-ChIP does not rely on formaldehyde fixation, which crosslinks primary amines such as those in lysine-rich histones [27]. Thus, we circumvent crosslinking of remodelers solely to their nearest nucleosome [17] and are able to directly map the interaction of remodelers with DNA. We and others have previously mapped transcription factors (TFs) on native chromatin from yeast and human cells [28]–[30], and we have shown similar recovery of MNase-protected DNA fragments from both total nuclei and solubilized chromatin from yeast without crosslinking [30]. Thus, it appears that formaldehyde crosslinking may be dispensable for the detection of protein-DNA interactions.

**Figure 1:**
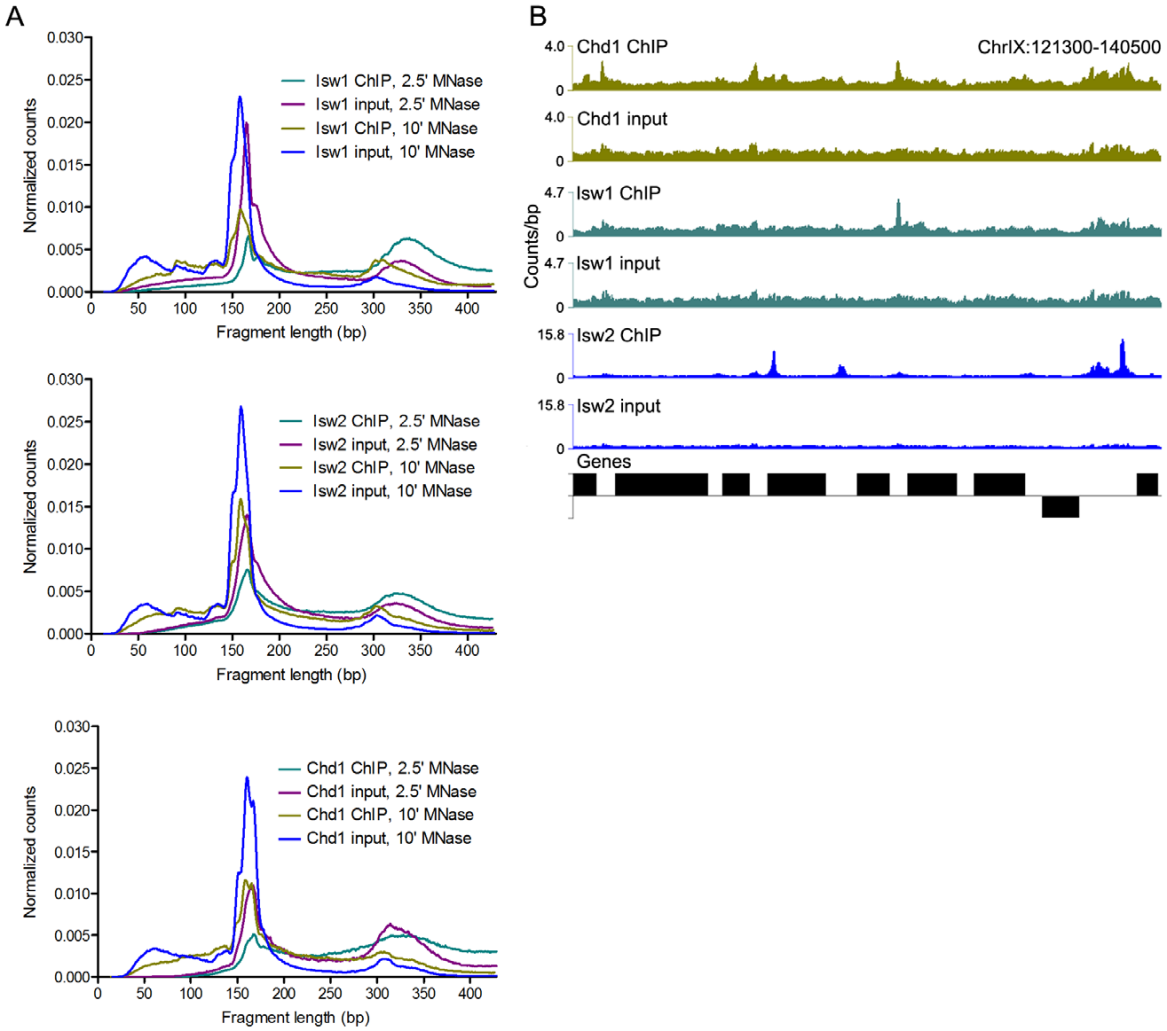
N-ChIP-seq localizes ISWI and CHD remodelers throughout the genome. (A) Size distributions of mapped paired-end 2.5′ and 10′ MNase-digested wild-type Isw1, Isw2, and Chd1 ChIP and input fragments. Slight variations in the amount of supernucleosomal (>251 bp) fragments are attributable to minor variation in the degree of MNase digestion for each sample, as evidenced by slight differences in the nucleosomal maxima for each sample. (B) Binding profiles for 10′ MNase-digested Isw1, Isw2, and Chd1 samples across a representative region of the genome. The number of paired-end reads overlapping each genomic position (counts/bp) is indicated on the Y-axis. See Figure S1 for additional remodeler binding profiles.

ChIP and input samples were prepared for sequencing using a modified protocol that recovers fragments as small as ∼25 bp, enabling base-pair resolution mapping of remodeler occupancy [30]. Samples treated with MNase for 2.5′ displayed a nucleosomal peak centered at ∼170 bp, which was shifted to ∼160 bp in the 10′ samples (Figure 1A). Examination of these size distributions indicated that ChIP samples were enriched for supernucleosomal DNA fragments relative to nucleosomes. To assess this observation systematically, we determined the area under the curve for nucleosomal (141–250 bp) and supernucleosomal (251–428 bp) fragments from each sample. ChIP samples, regardless of factor, displayed supernucleosomal/nucleosomal ratios 1.74–3.87 (2.5′ MNase, *P* = 0.009) and 2.26–2.76 (10′ MNase, *P* = 0.002) times greater than that seen in input samples (Table S2), suggesting that Isw1, Isw2, and Chd1 participate in the protection of large stretches of DNA.

**Figure 2:**
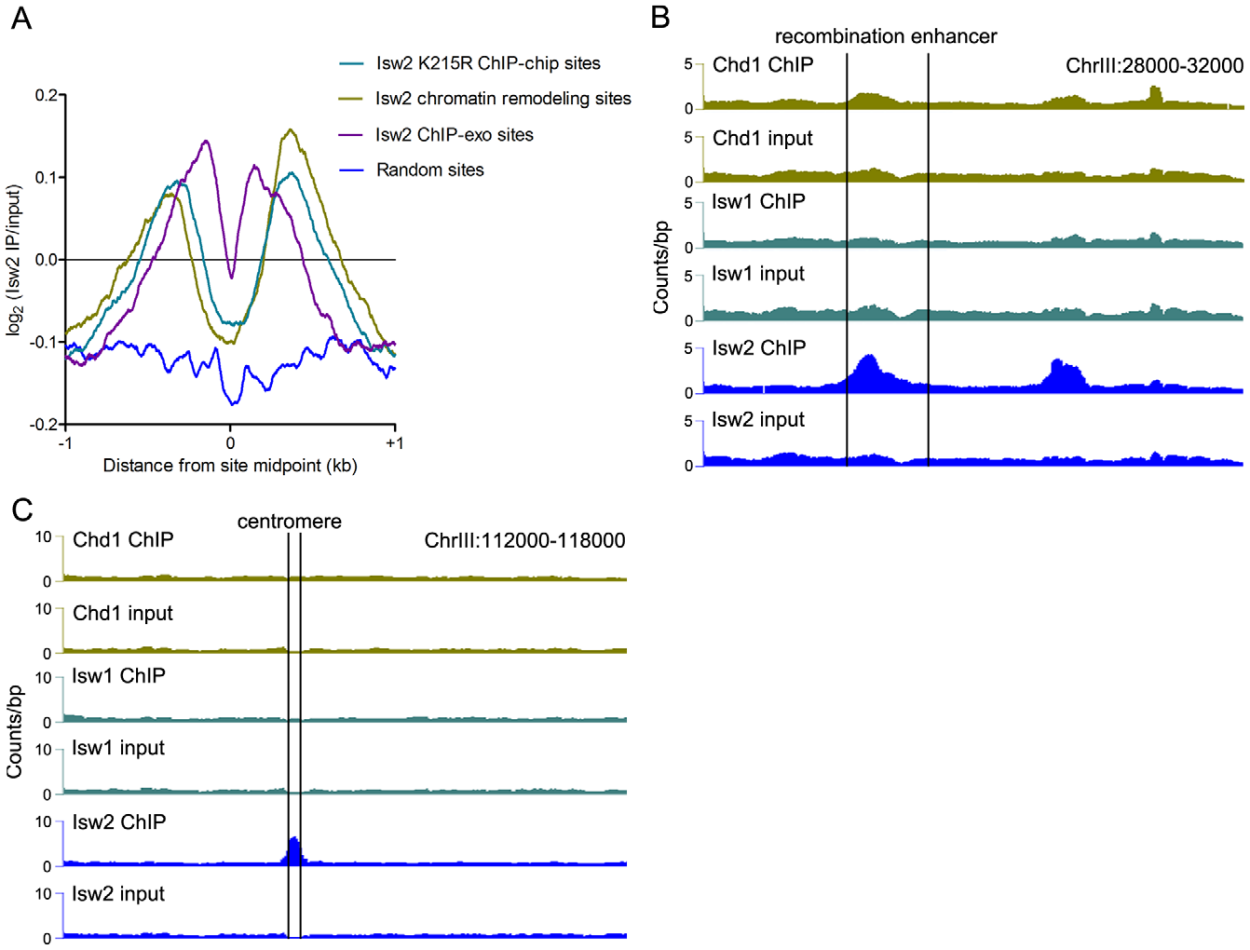
Comparison of Isw2 N-ChIP and X-ChIP data. (A) Aggregate profiles of log_2_(Isw2 IP/input) at sites bound by catalytically inactive (K227R) Isw2 in ChIP-chip experiments (2128 sites), sites with altered nucleosome positioning in an *isw2Δ* strain (1399 sites), sites bound by wild-type Isw2 in ChIP-exo experiments (1251 sites), and around random nucleosomes (1399 sites).Also shown are profiles of remodeler binding at the (B) chrIII recombination enhancer and (C) chrIII centromere (marked by vertical lines).

N-ChIP-seq revealed specific sites of enrichment for all three remodelers throughout the yeast genome (Figure 1B and Figure S1). We then tested if we could recapitulate known features of remodeler-genome association by N-ChIP-seq. We focused on Isw2, which has previously been characterized by genome-wide ChIP-chip [20], [31] and the recently developed ChIP-exo method [17]. ChIP-exo is a modification of the standard ChIP-seq protocol employing exonuclease digestion to improve the resolution of crosslinking ChIP-seq. Our N-ChIP-seq methodology is distinct from these techniques in that it does not rely on formaldehyde fixation, which also crosslinks primary amines to fix protein-protein interactions. We first obtained lists of Isw2 peaks determined by ChIP-chip [20] and ChIP-exo [17] as well as sites with altered nucleosome positioning in an *isw2Δ* strain [20]. We then determined the input-normalized N-ChIP-seq signal for each base pair in a 2-kb window centered on each ChIP or remodeling site and averaged the signal for each base pair within the window for each class of sites (Isw2 X-ChIP-chip, Isw2 ChIP-exo, and Isw2 remodeling) to generate average N-ChIP-seq profiles. We detected enrichment of Isw2 by N-ChIP-seq at sites bound by Isw2 in both X-ChIP-chip and ChIP-exo and sites of chromatin remodeling by Isw2 but not around randomly selected nucleosomes (Figure 2A). The two peaks on either side of the midpoint represent robust enrichment of Isw2 on either side of transcription factor binding sites. We note that the enrichment of Isw2 at these sites does not imply an effect on expression of nearby genes, only that our technique is able to replicate Isw2 binding sites previously determined by X-ChIP methodologies.

Sites of Isw2 action display high A+T content, which stiffens DNA and disfavors nucleosome formation [32]. We analyzed the bendability of DNA at sites occupied solely by Isw2 or by Isw1 and/or Chd1 without Isw2. Isw2-only N-ChIP-seq sites showed a reduction of ∼0.375 in average bendability compared to Isw1/Chd1 sites (*P* = 5.3×10^−41^). We also detected strong enrichment of Isw2 but little or no binding of Isw1 or Chd1 at the A+T rich yeast recombination enhancer (Figure 2B), as previously demonstrated [32]. We further noted enrichment of Isw2 at the chromosome III centromere, a ∼90% A+T sequence (Figure 2C). These results indicate that our N-ChIP-seq protocol faithfully captures known features of Isw2 genomic association.

### Isw2 preferentially binds centromeres

**Figure 3:**
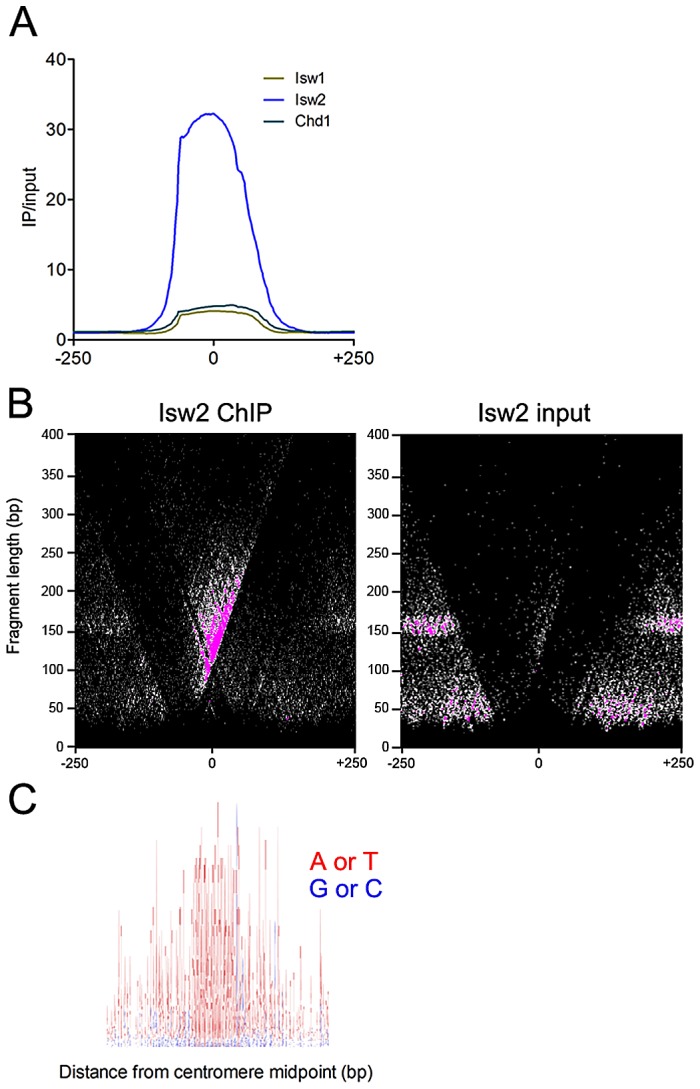
Isw2 associates with centromeres. (A) Aggregate plot of Isw1, Isw2, and Chd1 ChIP/input signal at all 16 yeast centromeres. (B) V-plot of Isw2 ChIP data for all 16 yeast centromeres showing enrichment of Isw2 to the CDEIII side of centromeres. (C) Sequence logo of all 16 yeast centromeres spanning 400 bp centered on the centromere midpoint. A+T are represented as red and G+C are represented as blue, demonstrating the high A+T content of centromeres. The binding of Isw2 to centromeres is thus consistent with its preference for association with regions of high A+T content. The sequence logo was generated with WebLogo (http://weblogo.berkeley.edu).

The enrichment of Isw2 at the chromosome III centromere led us to investigate Isw2 centromeric association in more detail. Aggregate analysis of remodeler signal at all 16 yeast centromeres revealed robust enrichment of Isw2, but not Isw1 or Chd1 (Figure 3A). To precisely delineate the regions of the centromere bound by Isw2, we employed V-plotting [30]. In a V-plot, the midpoint of each paired-end fragment is assigned a dot in two-dimensional space, wherein the X-axis value is the distance of the fragment midpoint from a defined genomic feature and the Y-axis value is the length of the fragment. V-plotting of Isw2 ChIP and input data revealed striking enrichment of Isw2 to the right of centromere midpoints, over the CDEII and CDEIII regions (Figure 3B). Analysis of centromeric sequence composition confirmed the A+T rich nature of yeast centromeres, further supporting the preference of Isw2 for A+T rich DNA (Figure 3C). The association of Isw2 with centromeres is consistent with previous X-ChIP results showing association of Isw2 with two yeast centromeres [33] as well as studies demonstrating a role for Isw2 in pericentromeric nucleosome dynamics [34] and centromeric association of the human ISWI-containing RSF complex [35].

### ISWI and CHD remodelers occupy extended linker DNA at transcription factor binding sites

**Figure 4:**
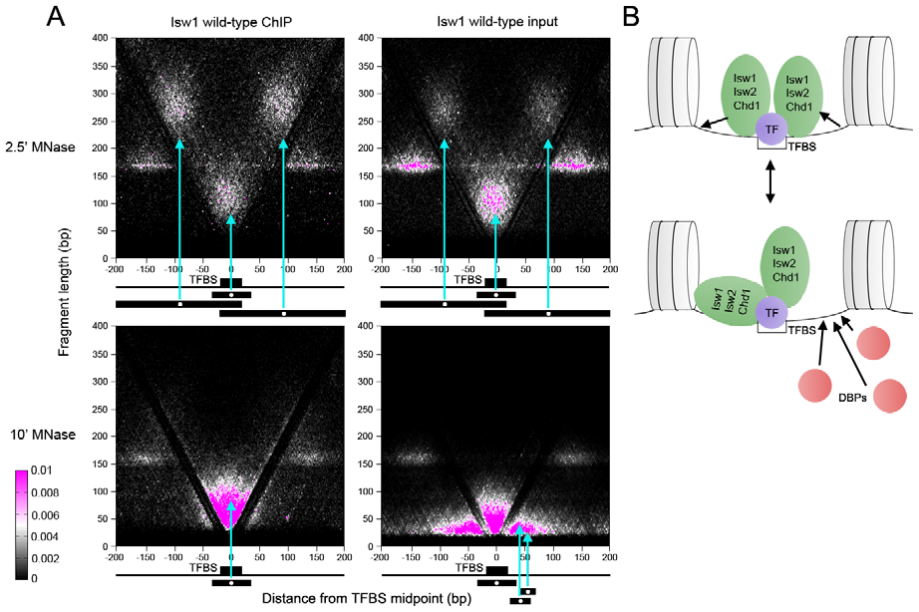
ISWI and CHD remodelers associate with TFBSs. (A) V-plots of wild-type Isw1 ChIP and soluble input chromatin at binding sites for the Abf1 TF after 2.5′ and 10′ MNase digestion. Flanking nucleosomes are visualized as well-defined dot clusters on either side of the TFBS. Example fragments contributing to the generation of various V-plot features are shown schematically below each plot. Cyan arrows point to the position of each fragment midpoint within the V-plot. Similar results were seen for wild-type Isw2 and Chd1 at Abf1 sites and for Isw1, Isw2, and Chd1 at Cbf1, Mbp1, and Reb1 binding sites (Figures S3 and S4). (B) Interpretive schematic of V-plot results. DBPs; DNA-binding proteins.

Previous work has demonstrated that sequence-specific TFs induce nucleosome depletion upon binding [36]–[41], presumably by exposing stretches of linker DNA. We therefore hypothesized that the enrichment of supernucleosomal DNA fragments in ChIP samples might reflect remodeler association with the extended linker DNA flanking binding sites for nucleosome-phasing TFs. We assessed transcription factor binding site (TFBS) occupancy of Isw1, Isw2, and Chd1 using V-plotting. We first analyzed remodeler occupancy at binding sites for the Abf1 TF. Consistent with our previous data [30], well-phased flanking nucleosomes were observed at Abf1 sites as discrete dot clusters to either side of the TFBS (Figure 4A and Figure S2A).

In samples treated with MNase for 2.5′, we noted robust enrichment of supernucleosomal fragments spanning ∼250–300 bp and centered between the TFBS and each flanking nucleosome in ChIP, and to a lesser extent, input samples. These supernucleosomal fragments represent continuous protection of DNA from the flanking nucleosome to the opposite side of the TFBS, with remodeler complexes occupying the linker DNA spanning the nucleosome and TFBS. Additionally, supernucleosomal fragments were depleted from the more heavily digested 10′ samples, while smaller fragments flanking both sides of the TFBS were seen (Figure 4A and Figure S2A), suggesting that remodeler-linker DNA associations display varying degrees of stability.

As observed previously, clusters of subnucleosomal particles flank TFBSs in our input chromatin samples [30]. These particles were not observed in remodeler ChIP V-plots (Figure 4A and Figure S2A), indicating that they are not remodelers but rather small DNA-binding proteins. Fragment sizes in the central protected region of the ChIP V-plots extended ∼30 bp higher than in the input V-plots, perhaps indicating stable association of remodelers closer to the TFBS (Figure 4A and Figure S2A). We also generated V-plots of 2.5′ and 10′ MNase ChIP and input data using Cbf1, Mbp1, and Reb1 binding sites, which, like Abf1 sites, display well-phased flanking nucleosomes (Figure S2B–S2D). In each case, similar V-plots were seen for Isw1, Isw2, and Chd1.

These V-plot observations suggest that remodelers bind to regions of extended linker DNA created by TF-induced nucleosome depletion at TFBSs and protect variable amounts of flanking DNA (Figure 4B, top). A remodeler molecule then occupies an extended region up to the flanking nucleosome to generate the large supernucleosomal fragment clusters observed after 2.5′ MNase digestion. Unprotected linker DNA on the non-remodeler protected side of the TF could then be occupied by DNA-binding proteins (Figure 4B, bottom). Depletion of supernucleosomal fragments in the 10′ MNase-digested samples suggests that remodelers dynamically protect linker DNA on one or the other side of the TFBS. This association of Isw1, Isw2, and Chd1 with substantial extranucleosomal DNA is consistent with *in vitro* studies in yeast, *Drosophila*, and humans [6]–[10], [42], [43].

### Remodeler association with transcription factor binding sites is ATP-independent

**Figure 5:**
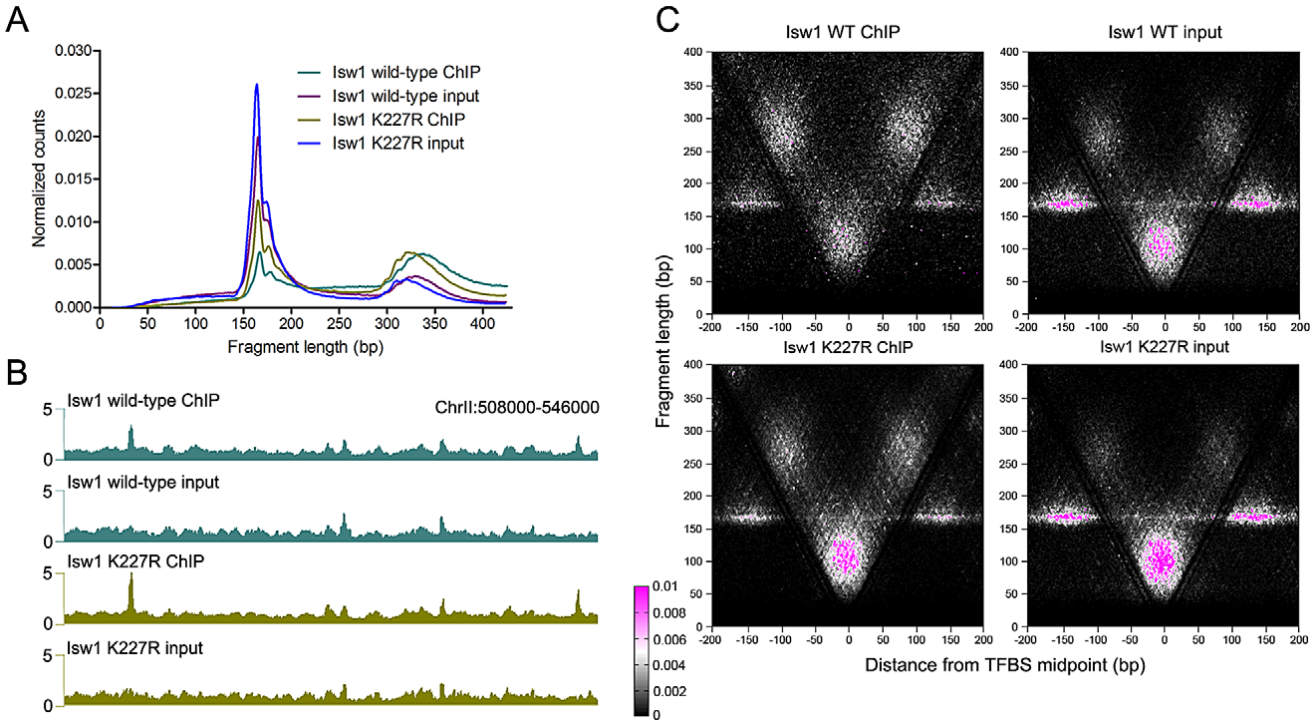
ISWI and CHD remodeler association with TFBSs is ATP-independent. (A) Size distributions of mapped paired-end 2.5′ MNase-digested wild-type and catalytically inactive K227R Isw1 ChIP and input fragments. Similar profiles were seen for Isw2 K215R and Chd1 K407R (Figure S4). (B) Profiles of wild-type and K227R Isw1 binding along a representative segment of the genome. (C) V-plots of wild-type and K227R Isw1 ChIP and soluble input chromatin at binding sites for the Reb1 TF, determined by ChIP-exo, after 2.5′ MNase digestion. The overall fragment size in the Isw1 K227R ChIP and input samples is slightly reduced when compared to wild-type, indicative of technical variation in MNase digestion. Similar results were seen for K227R Isw1 and catalytically inactive Isw2 (K215R) and Chd1 (K407R) at Abf1 and other TFBSs (Figures S4 and S6).

We next asked whether remodeler inactivation would alter binding around TFBSs. Surprisingly, elimination of remodeler catalytic activity via a lysine-to-arginine substitution in the conserved GXGKT ATP-binding motif [21], [44] did not substantially affect the size distribution of mapped fragments (Figure 5A and Figure S4) or the genomic binding profiles of remodelers (Figure 5B). We assessed the effect of remodeler catalytic inactivation by V-plotting wild-type and catalytically inactive (K227R) ChIP and input data at sites for the Reb1 TF generated by ChIP-exo [45], which also allowed us to assess V-plot patterns using an independent set of binding sites. V-plot patterns generated with ChIP-exo Reb1 sites were nearly indistinguishable from those created using Reb1 ChIP-chip sites (Figure 5C, Figure S3, Figure S5). Catalytic inactivation of remodelers also had no noticeable effect on V-plot patterns at TFBSs (Figure 5C, Figure S3, and Figure S5), implying that remodeler association with TFBSs is ATP-independent.

### ISWI and CHD remodelers preferentially bind NDRs

**Figure 6:**
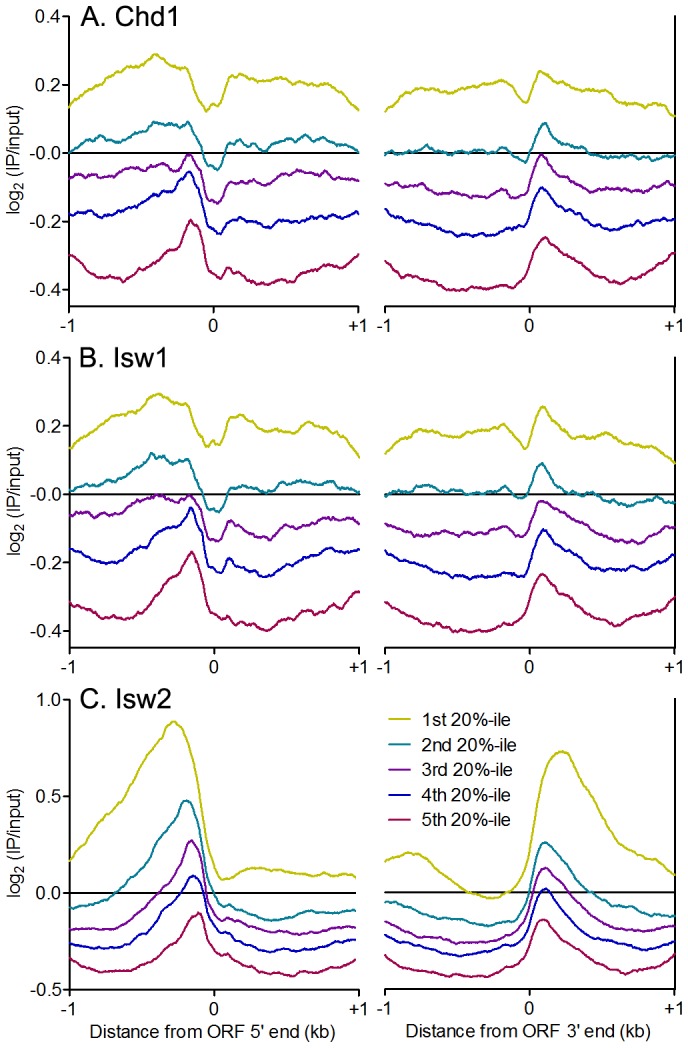
ISWI and CHD remodelers bind NDRs. Aggregate plots of log_2_(IP/input) signal ±1 kb of verified ORF 5′ and 3′ ends for (A) Chd1, (B) Isw1, and (C) Isw2 ranked separated into quintiles by average remodeler signal.

The sharp patterns of phased nucleosomes on either side of binding sites for Abf1, Reb1 and other TFs is consistent with their known role in creating NDRs that lead to transcriptional activation [46]. Isw1and Chd1 position nucleosomes within gene bodies *in vivo*, while Isw2 generally positions nucleosomes flanking NDRs, preventing directional nucleosome shifting within gene bodies [16]–[21]. We therefore asked whether there is a relationship between remodeler occupancy and dynamics around individual TFBSs and features of adjacent genes. Consistent with their known functions, we observed enrichment of Chd1 and Isw1 within gene bodies (Figure 6A–6B), while Isw2 showed robust enrichment at NDRs, where TFBSs are generally located in yeast, with little gene body binding (Figure 6C). Strikingly, equal or slightly greater enrichment than that seen in gene bodies for Chd1 and Isw1 was observed at NDRs at the 5′ and 3′ ends of verified ORFs (Figure 6A–6B). We also detected Isw1 association with 5′ NDRs after adding a formaldehyde crosslinking step to our N-ChIP-seq protocol (Figure S6), indicating that remodeler-NDR association is not due to opportunistic, nonspecific interactions of remodelers with free DNA during chromatin preparation and immunoprecipitation. We assessed the association of remodeler binding with NDR size, reasoning that if remodeler-NDR interactions were simply due to the presence of large regions of naked DNA in the chromatin preparation, larger NDRs would display greater remodeler association. No such correlations were observed (Isw1 *R^2^* = 0.0339; Isw2 *R^2^* = 0.0397; Chd1 *R^2^* = 0.0519), further arguing against nonspecific association of remodelers with free DNA during our experiments.

Our Isw2 N-ChIP-seq results are somewhat different from those generated using ChIP-exo. We found Isw2 to be enriched upstream of ORF 5′ ends to a distance of nearly 1 kb in some cases (Figure 6C), whereas ChIP-exo showed very discrete localization of Isw2 to the TSS, immediately adjacent to the +1 nucleosome [17]. The differences between our N-ChIP-seq results and those of the ChIP-exo study may be explained by the crosslinking of Isw2 to the +1 nucleosome in ChIP-exo. Isw2 that is not adjacent to a nucleosome is less likely to be crosslinked and more likely to be lost during subsequent solubilization of chromatin. Our results indicate that there is at least some transient interaction between Isw2 and the +1 nucleosome, and we posit that this is the fraction of Isw2 previously mapped by ChIP-exo.

### ISWI and CHD remodeler association with gene bodies is associated with transcription-coupled nucleosome turnover

Our results demonstrate that yeast ISWI and CHD remodelers associate not only with gene bodies, where their effects are seen, but also with regions of extended linker DNA flanking TFBSs within NDRs. In the case of Isw2, robust binding to linker DNA within NDRs is consistent both with its *in vitro* preference for substantial extranucleosomal DNA and its action positioning nucleosomes adjacent to NDRs [7]–[9], [20]. However, while the binding of Chd1 and Isw1 to linker DNA within NDRs is in line with their *in vitro* association with significant amounts of linker DNA [6], [10], it appears at odds with their function within gene bodies [16]–[19], [21]. To reconcile the robust occupancy of Isw1 and Chd1 around NDRs with their function within gene bodies, we suggest that TFs generate regions of nucleosome depletion, which expose extended linker DNA and are thus favorable for remodeler-chromatin association. Transcriptional elongation then disrupts or evicts nucleosomes, extending regions of linker DNA within gene bodies and enabling efficient remodeling of the remaining gene-body nucleosomes and/or newly deposited nucleosomes by Isw1 and Chd1, while Isw2 simultaneously positions NDR-flanking nucleosomes to prevent directional shifting of gene-body nucleosomes, which could lead to harmful cryptic transcription [20].

**Figure 7:**
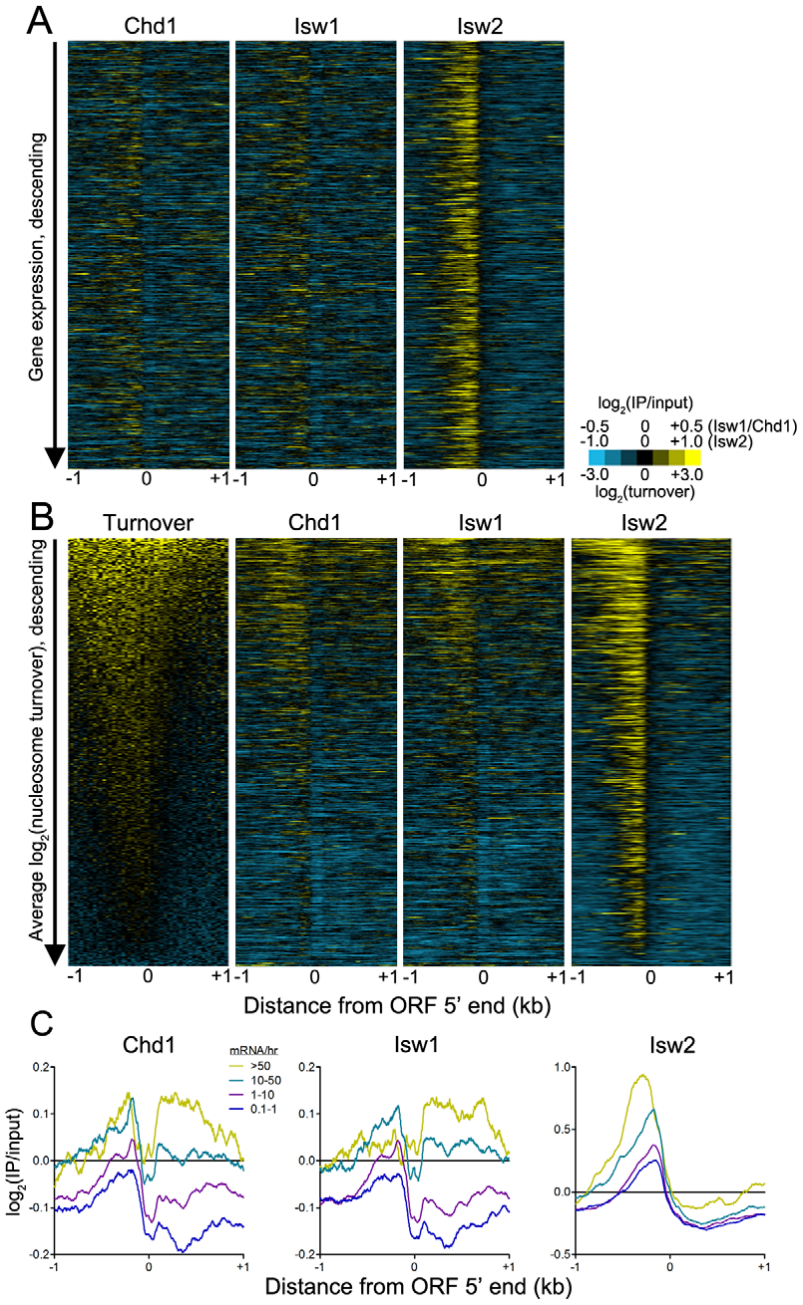
ISWI and CHD remodeler binding is positively associated with histone turnover and transcription rate. (A) Heat maps of log_2_(IP/input) signal for Chd1, Isw1, and Isw2 ±1 kb of verified ORF 5′ ranked descending by gene expression. (B) Heat maps of log_2_(nucleosome turnover) and log_2_(IP/input) signal for Chd1, Isw1, and Isw2 ±1 kb of verified ORF 5′ ranked descending by average nucleosome turnover across the entire 2-kb window. (C) Aggregate plots of Chd1, Isw1, and Isw2 log_2_(IP/input) signal ±1 kb of verified ORF 5′ ends, separated by transcription rate in mRNA/hr [47].

This model predicts that Chd1 and Isw1 association with gene bodies depends on transcription rate. As such, we would not expect to observe an association between remodeler binding and steady-state expression levels, as infrequently transcribed genes may yield mRNAs with long half-lives and vice versa [47]. Indeed, we observed no correlation between remodeler occupancy and steady-state gene expression (Figure 7A). Given that transcription is associated with histone turnover [48]–[52], we hypothesized that there might be a relationship between remodeler association and turnover. High turnover within ORF 5′ ends was postulated to reflect a requirement to maintain nucleosome depletion at promoters [51], which is achieved, at least in part, by TFs such as Abf1 and Reb1 and the SWI/SNF-family remodeler RSC [39], [41]. Comparison of histone turnover data with remodeler N-ChIP-seq data revealed a positive association between binding of remodelers to ORF 5′ ends and gene bodies and nucleosome turnover (Figure 7B). We also compared remodeler binding with transcription rate and found that highly transcribed genes were more highly bound by Isw1, Isw2, and Chd1 (Figure 7C). Taken together, these data suggest that Isw1 and Chd1 binding within gene bodies displaying high nucleosome turnover is a consequence of transcriptional elongation. While Isw2 generally does not bind gene bodies, its loss affects gene body nucleosomes, which shift to their thermodynamically favored positions in its absence [20]. The increased binding of Isw2 to the 5′ NDRs of highly transcribed genes may therefore reflect the increased nucleosome disruption caused by transcriptional elongation and, consequently, the increased requirement for the positioning activity of Isw2.

What is the relationship of ISWI and CHD remodelers to transcription? Previous work has demonstrated that simultaneous deletion of Isw1, Isw2, and Chd1 in yeast does not alter promoter nucleosome occupancy or positioning [16], indicating that these remodelers are unlikely to influence RNA polymerase II binding to promoters. Strikingly, this same work showed that loss of Isw1, Isw2, and Chd1 had relatively little effect on gene transcription, despite causing massive disorganization of gene body chromatin structure. These findings suggested that the major role of ISWI and CHD remodelers, and gene body chromatin organization in general, is to prevent cryptic transcription within gene bodies [20], [53]. In the case of Chd1 deletion, cryptic transcription is linked to increased gene body histone turnover, indicating a role for Chd1 in suppressing histone turnover [53]. This result is also consistent with our finding that Chd1 binding is associated with nucleosome turnover, as regions displaying high turnover would both provide exposed DNA for Chd1 to bind and require Chd1 to suppress excessive histone turnover and subsequent cryptic transcription. Furthermore, loss of Chd1 impairs post-elongation nucleosome reassembly [54]. In light of our data and these previous results, we propose the following model for the relationship of Chd1 to transcription. A round of transcriptional initiation and elongation occurs, displacing or evicting nucleosomes. Chd1, either by interacting with the elongation machinery or following in the wake of RNA polymerase II, can then scan for regions of exposed linker DNA to which it can bind and suppress turnover and, by extension, cryptic transcription. In support of this possibility, Chd1 has been shown to genetically and physically interact with the transcription machinery in several species [55]–[58], and Isw1 and Isw2 genetically interact with elongation factors in yeast [59].

It is estimated that there is sufficient Isw2 for a molecule be bound every 2–5 kb of DNA, with a similar abundance for Chd1 and twice that amount for Isw1 (T.T., unpublished). Additionally, high salt is required for remodeler extraction from cells, indicating that they are bound to chromatin [44], [60]. Therefore, as much as 5–10% of the yeast genome, perhaps most NDRs, may be occupied by Isw1, Isw2, and Chd1. *Drosophila* ISWI is similarly abundant [61] and multiple Chd1-related remodelers are highly abundant and active in many higher eukaryotes [62]–[64], suggesting that the binding of ISWI and CHD remodelers at NDRs and their subsequent action downstream is an ancestral mechanism for chromatin structure maintenance.

## Materials and Methods

### Yeast methods

Yeast cells were grown in YPD and harvested at OD_600_ = 0.6–0.8. Mutation of Chd1 K407 was carried out as follows. Primers F-5′- GGTACCCTTGGGAGAATGCCACAGAT -3′, containing a KpnI site (underlined) and R-5′- ATCGATCTTCGTCAACGGCCATAAAT -3′, containing a ClaI site (underlined) were used to amplify nt 961–1543 of *CHD1*. The amplified fragment was TOPO-cloned into pCR2.1. pCR2.1-*CHD1*-961–1543 was digested with KpnI and ClaI and the *CHD1* fragment was ligated into pRS406 and mutagenized with the QuikChange kit (Agilent). Mutation was confirmed by sequencing and introduced a strain harboring a Chd1-3xFLAG allele via the pop-in/pop-out method [65]. Yeast strains used in this study are given in Table S1.

### Nuclear isolation and chromatin preparation

Nuclei were isolated from yeast cells and stored at −80°C in 5 ml sorbitol-PIPES-calcium (SPC) buffer as described [66]. For X-ChIP, cells were fixed with 1/10 culture volume buffered formaldehyde solution (50 mM HEPES-KOH, 100 mM NaCl, 1 mM EDTA, 0.5 mM EGTA, 11% formaldehyde) for 15 min at RT with gentle rotation and quenched with 18 ml/100 ml culture volume 2.5 M glycine prior to nuclear isolation. Upon thawing, SPC buffer was supplemented with 1 mM phenylmethanesulfonylfluoride (PMSF), 10 µg/ml LPC [66], and 2 mM CaCl_2_. Chromatin was digested with MNase for 2.5 or 10 min and digestion was stopped by the addition of EDTA to 10 mM. MNase-treated nuclei were then passed 4 times through a 20-gauge needle and 4 times through a 26-gauge needle [30], [67] and centrifuged at 10,000 RPM for 10 min. The supernatant (S1) was held on ice. The pellet was resuspended in 5 ml extraction buffer (10 mM phosphate buffer pH 7.4, 70 mM NaCl, 0.75 mM EDTA, 0.1% Triton X-100) supplemented with 1 mM PMSF and 10 µg/ml LPC and rocked on a nutator for 4 hours at 4°C. Samples were centrifuged at 13,000 RPM for 15 min at 4°C and the supernatant (S2) was saved. Triton X-100 was added to S1 to a final concentration of 0.1%, S1 and S2 were combined, and the salt concentration of the combined 10 ml chromatin solution was adjusted to 80 mM with NaCl.

### ChIP

100 µl of each chromatin sample was saved as input. 100 µl of FLAG M2 magnetic beads (Sigma M8823, lot #041M6135) were used for all remodeler-FLAG immunoprecipitations (IPs). Beads were washed 3 times with PBS/0.5% BSA and added to the chromatin samples. IPs were incubated overnight at 4°C with end-over-end rotation. Beads were washed 3 times with IP buffer (10 mM phosphate buffer, pH 7.4, 0.75 mM EDTA, 70 mM NaCl) supplemented with 10 µg/ml LPC and 1 mM PMSF and resuspended in 400 µl IP buffer. 1/50^th^ volumes of 5 M NaCl and 0.5 mM EDTA were added to IP and input samples and RNA was digested with 1 µg RNase A for 10 min at 37°C. SDS was added to IP and input samples to a concentration of 0.5% and protein was digested with 80 µg Proteinase K for 20 min at 65°C. 20 µg glycogen was added to each IP and input sample followed by extraction with 1 volume phenol/chloroform/isoamyl alcohol. DNA was precipitated with 2 volumes 100% ethanol for 30 min at −80°C and washed twice with 500 µl 100% ethanol. Precipitated DNA was resuspended in 20 µl 0.1X TE buffer, pH 8.0 and quantified by PicoGreen [30].

X-ChIP was performed as above with the following modifications. In addition to washing in IP buffer, IPs were washed twice with TSE I (0.1% SDS, 1% Triton X-100, 2 mM EDTA, 20 mM Tris pH 8.0, 150 mM NaCl) and twice with TSE II (0.1% SDS, 1% Triton X-100, 2 mM EDTA, 20 mM Tris pH 8.0, 500 mM NaCl). Elution and crosslink reversal was performed overnight at 65°C in elution buffer (50 mM Tris pH 8.0, 10 mM EDTA, 1% SDS). Following elution, 1 volume of TE buffer pH 8.0 was added to each sample to dilute the SDS to 0.5% for RNase A and proteinase K treatment as above.

### Sequencing library preparation

Sequencing libraries were prepared according to our previously described modification of the standard Illumina protocol [30]. Libraries were sequenced for 25 cycles in paired-end mode on the Illumina HiSeq 2000 platform at the Fred Hutchinson Cancer Research Center Genomics Shared Resource.

### Data analysis

Paired-end sequencing data were processed and aligned to the V64/SacCer3 genome build with Novoalign (Novocraft; http://www.novocraft.com) and processed as described [30], [68]. Numbers of paired-end fragments mapped to the genome for each sample are given in Table S2. Size distributions were normalized such that the area under the curve for each sample was equal to 1. V-plots were generated with gnuplot (http://www.gnuplot.info). The V-plot scale represents the percentage of fragments used in constructing each V-plot contained in each bin (pixel) of the plot (160,400 bins total).

Isw2 K215R ChIP-chip binding sites, sites of chromatin remodeling by Isw2, and random sites were obtained from Whitehouse et al. [20] and Isw2 ChIP-exo binding sites were obtained from Yen et al. [17]. The log_2_(IP/input) N-ChIP-seq signal for each base pair ±1 kb of the midpoint of each Isw2 K215R-bound region, site of Isw2 remodeling, Isw2 ChIP-exo site, and random site was determined as above and averaged to generate aggregate plots.

For DNA bendability analysis, 29 100 bp regions bound by Isw2 without Isw1 and/or Chd1 and 29 100 bp regions bound by Isw1 and/or Chd1 without Isw2 were obtained. Regions were analyzed using the bend.it server [69] with a window size of 39 bp. Bendability values for each position within the window were averaged and the two classes of sites were compared by t-test.

The SGD verified ORFs list was used to determine transcription unit length and ORF 5′ and 3′ end coordinates. For heatmaps, the log_2_(IP/input) for each base pair ±1 kb of each ORF 5′ or 3′ coordinate was determined using a custom Perl script [70]. Heatmaps were visualized with Java TreeView [71]. All heatmaps were generated using 10′ MNase data and are oriented such that the direction of transcription for all genes is to the right. Expression data were obtained from GEO (GSM552681) [72]. Nucleosome turnover data were obtained from Dion et al. [51]. Nucleosome turnover signal was obtained as above, but using 40 bp windows due to the lower resolution of these data. Transcription rates were obtained from Holstege et al. [47]. Correlations between remodeler signal and NDR size were generated using a previously annotated list of NDRs [73] and the maximum log_2_(IP/input) N-ChIP-seq signal within each NDR.

### Data availability

Sequencing data generated in this publication have been deposited with GEO (GSE39331).

## Supporting Information

Figure S1 Additional Isw1, Isw2, and Chd1 N-ChIP-seq profiles. Signal tracks of Isw1, Isw2, and Chd1 binding in representative regions of the genome. Counts/bp is indicated on the Y-axis.
(TIF)

Figure S2 V-plots of wild-type Isw1, Isw2 and Chd1 ChIP and input data at Abf1, Cbf1, Mbp1, and Reb1 sites. Note the discrete lower size limit of flanking nucleosomes at ∼147 bp in 10′ MNase-treated samples, indicating that these nucleosomes are tightly wrapped.
(TIF)

Figure S3 V-plots of wild-type and catalytically inactive Isw1, Isw2, and Chd1 ChIP and input data at ChIP-exo-defined Reb1 binding sites. Binding sites are derived from the data of Rhee and Pugh [45].
(TIF)

Figure S4 Loss of remodeler catalytic activity does not alter remodeler fragment size distributions. Size distributions of mapped paired-end 2.5′ MNase-digested wild-type and K215R Isw2 and wild-type and K407R Chd1 ChIP and input fragments.
(TIF)

Figure S5 V-plots of catalytically inactive Isw1, Isw2 and Chd1 ChIP and input data at Abf1, Cbf1, Mbp1, and Reb1 sites.
(TIF)

Figure S6 Isw1 association with 5′ NDRs is captured by X-ChIP-seq. Heatmaps of log_2_(Isw1 IP/input) native and crosslinked 2.5′ MNase signal ±1 kb of verified ORF 5′ ends ranked descending by gene expression level.
(TIF)

Table S1 Yeast strains used in this study. All strains are derived from W1588-4C, which is isogenic to W303-1A except that a weak *RAD5* mutation is repaired [74].
(DOC)

Table S2 Numbers of mapped pairs and supernucleosomal/nucleosomal AUC ratios for sequenced samples. ChIP/input is the ChIP supernucleosomal/nucleosomal AUC ratio divided by the input supernucleosomal/nucleosomal AUC ratio.
(DOC)
