# Supplementary figures and images for: ISWI and CHD Chromatin Remodelers Bind Promoters but Act in Gene Bodies
Source: PLoS Genet. 2013 Feb 28;9(2):e1003317. doi: 10.1371/journal.pgen.1003317 (PMC3585014; doi:10.1371/journal.pgen.1003317)

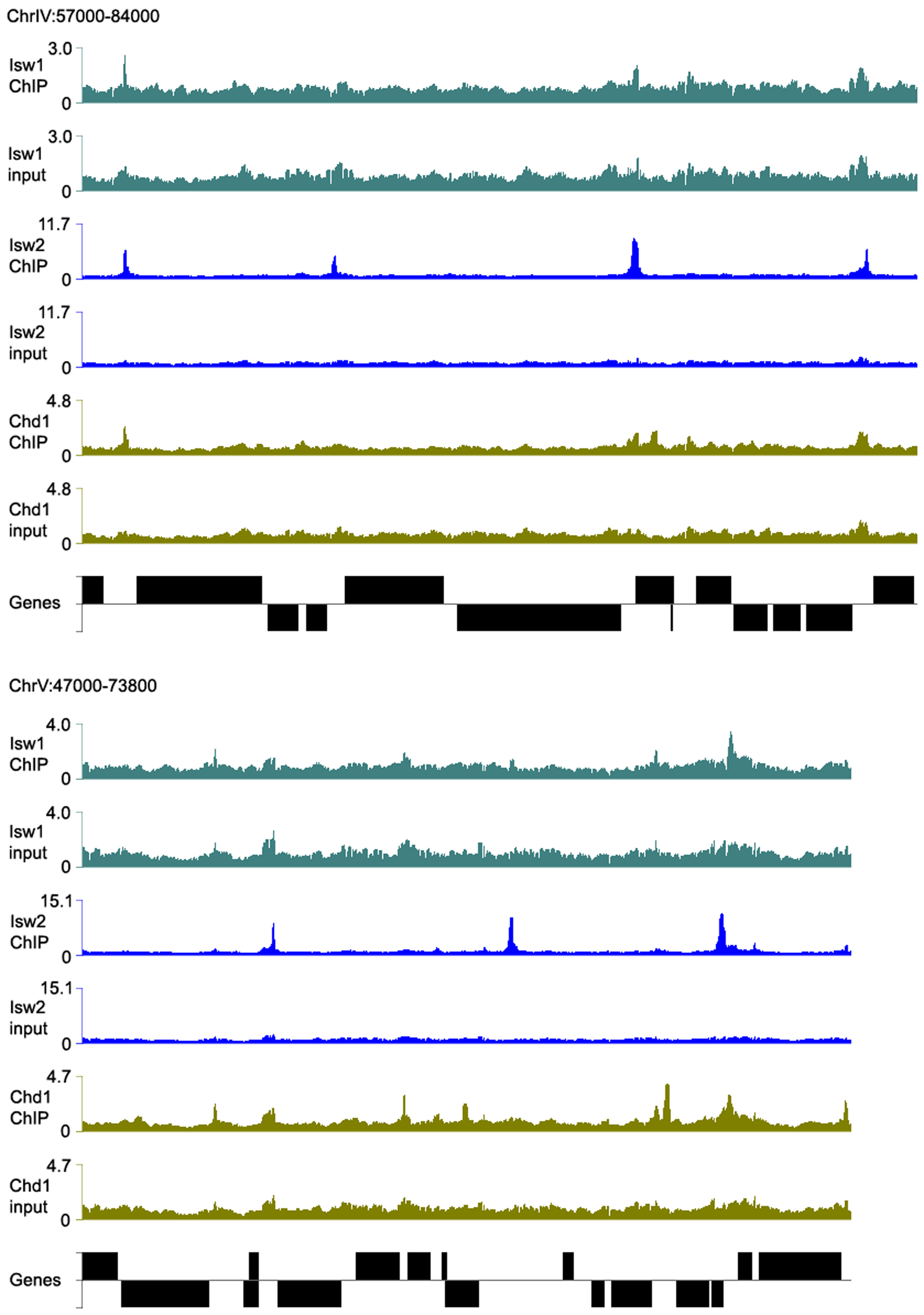

Supplement: Figure S1 — Additional Isw1, Isw2, and Chd1 N-ChIP-seq profiles. Signal tracks of Isw1, Isw2, and Chd1 binding in representative regions of the genome. Counts/bp is indicated on the Y-axis. (TIF) [file pgen.1003317.s001.tif]

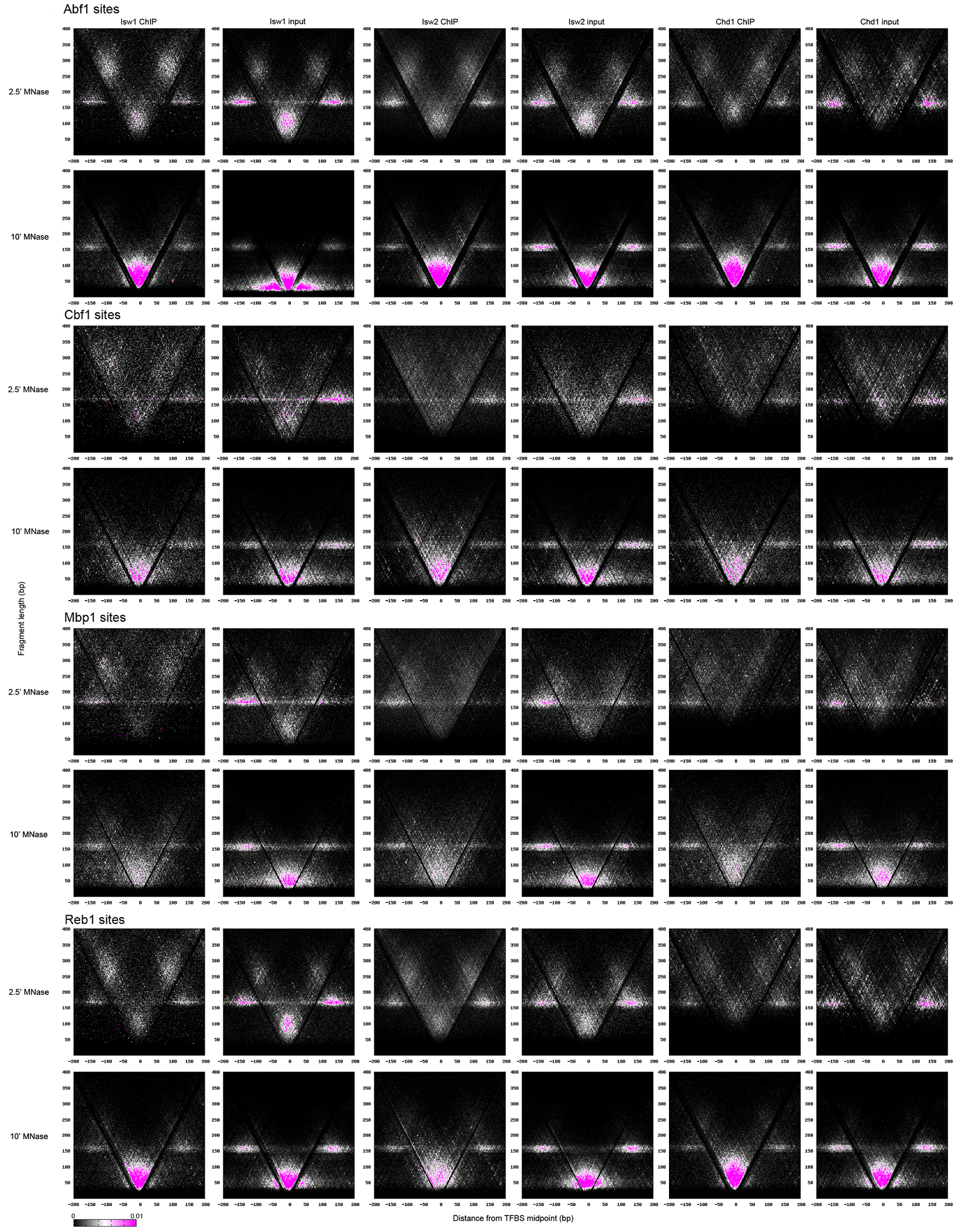

Supplement: Figure S2 — V-plots of wild-type Isw1, Isw2 and Chd1 ChIP and input data at Abf1, Cbf1, Mbp1, and Reb1 sites. Note the discrete lower size limit of flanking nucleosomes at ∼147 bp in 10′ MNase-treated samples, indicating that these nucleosomes are tightly wrapped. (TIF) [file pgen.1003317.s002.tif]

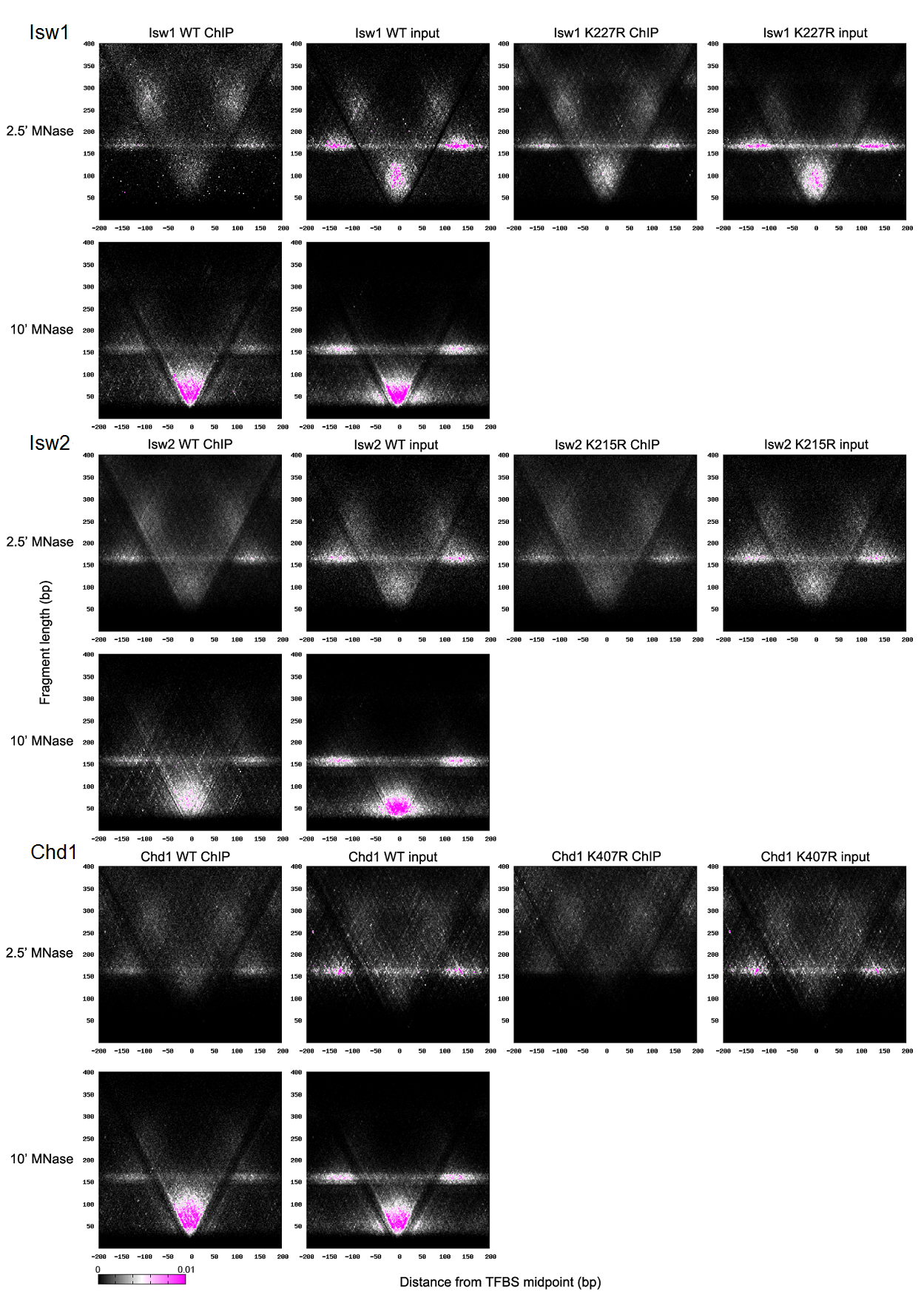

Supplement: Figure S3 — V-plots of wild-type and catalytically inactive Isw1, Isw2, and Chd1 ChIP and input data at ChIP-exo-defined Reb1 binding sites. Binding sites are derived from the data of Rhee and Pugh [45]. (TIF) [file pgen.1003317.s003.tif]

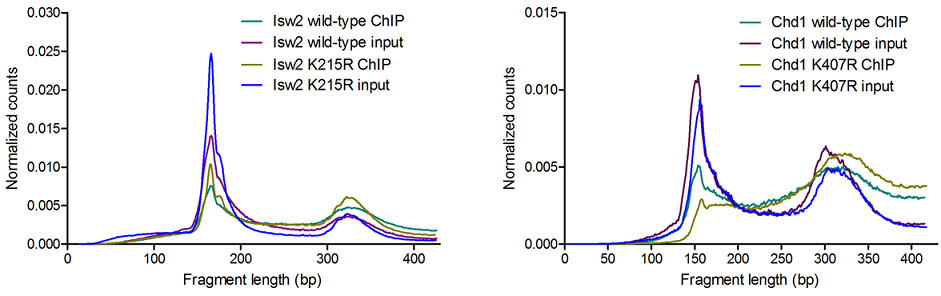

Supplement: Figure S4 — Loss of remodeler catalytic activity does not alter remodeler fragment size distributions. Size distributions of mapped paired-end 2.5′ MNase-digested wild-type and K215R Isw2 and wild-type and K407R Chd1 ChIP and input fragments. (TIF) [file pgen.1003317.s004.tif]

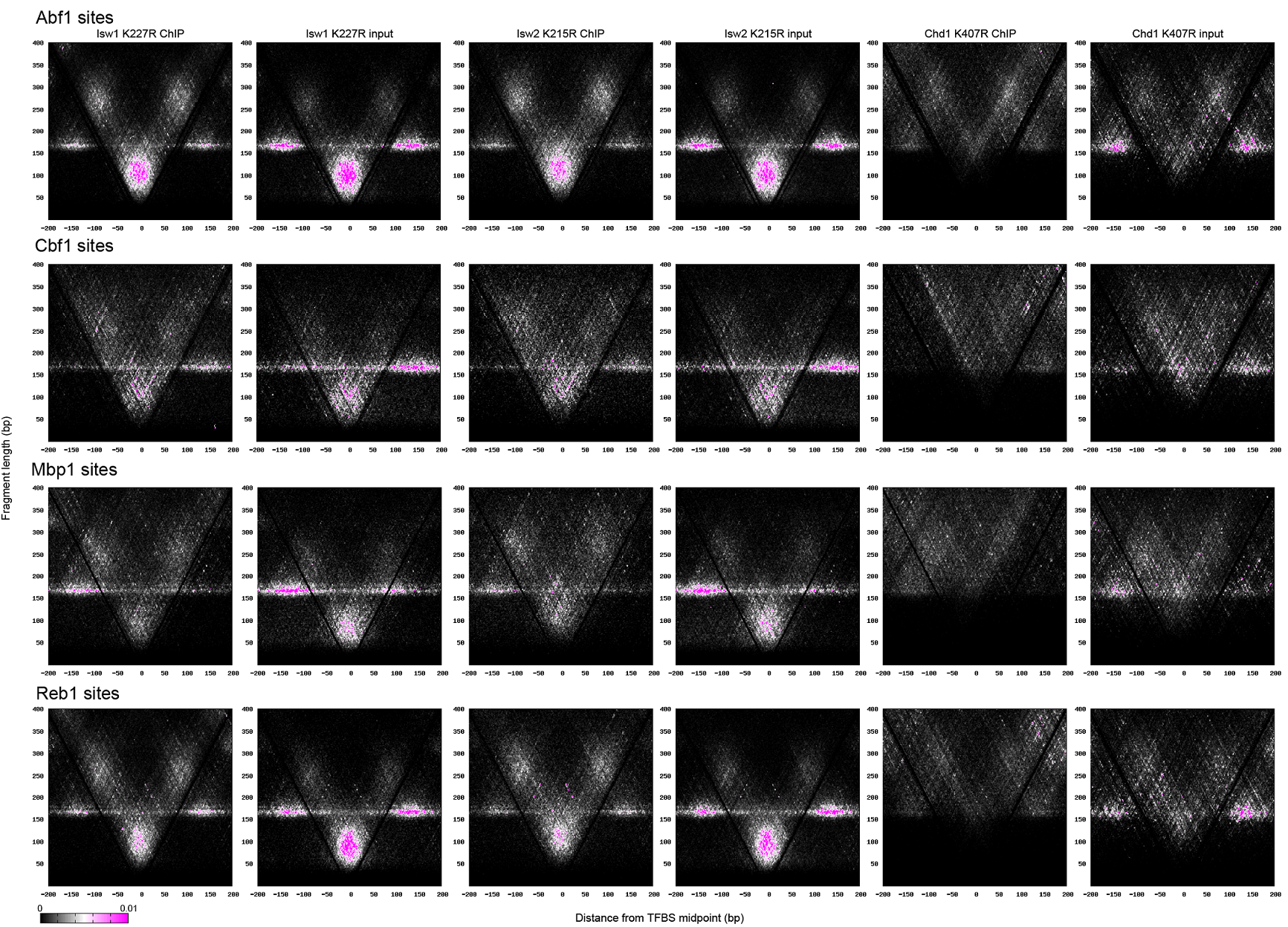

Supplement: Figure S5 — V-plots of catalytically inactive Isw1, Isw2 and Chd1 ChIP and input data at Abf1, Cbf1, Mbp1, and Reb1 sites. (TIF) [file pgen.1003317.s005.tif]

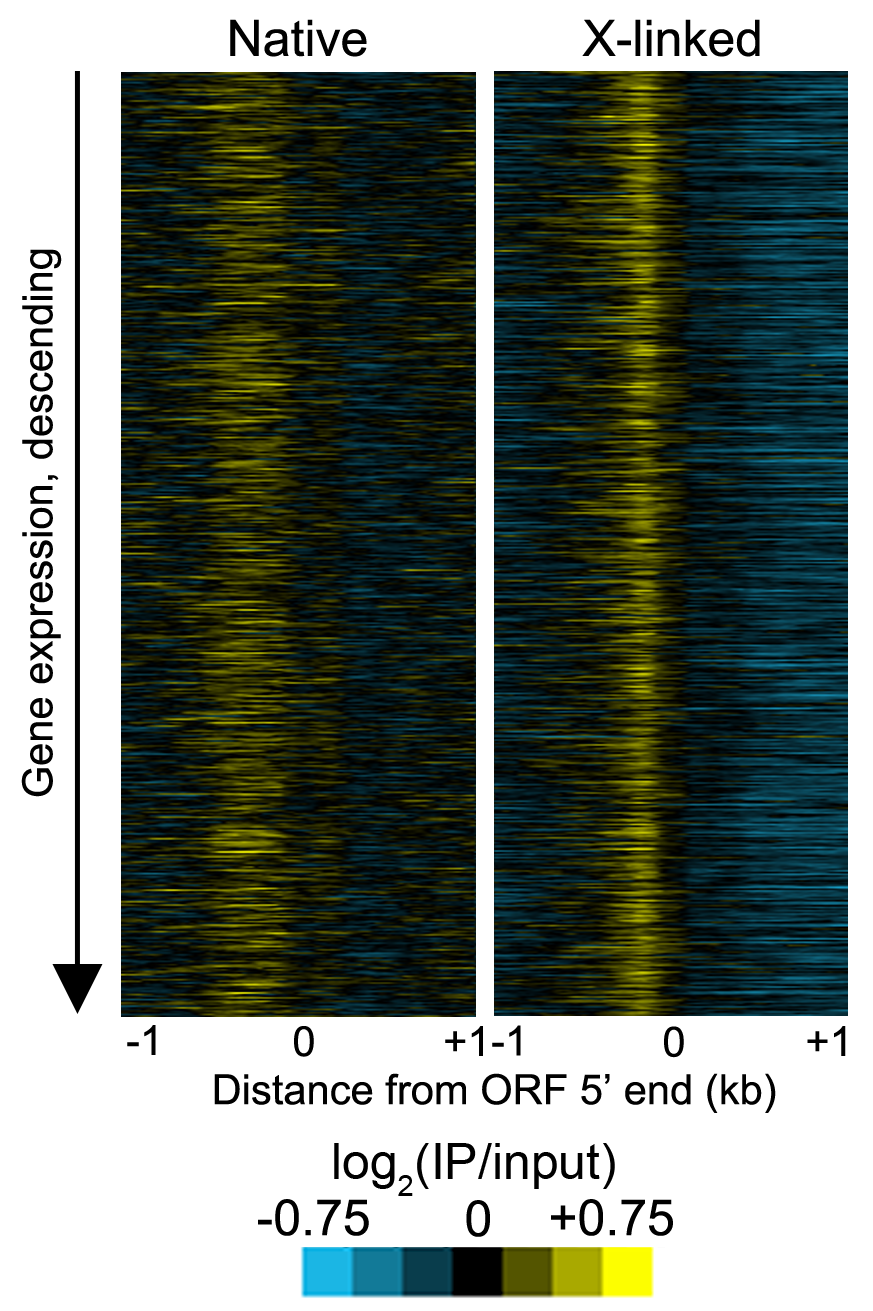

Supplement: Figure S6 — Isw1 association with 5′ NDRs is captured by X-ChIP-seq. Heatmaps of log2(Isw1 IP/input) native and crosslinked 2.5′ MNase signal ±1 kb of verified ORF 5′ ends ranked descending by gene expression level. (TIF) [file pgen.1003317.s006.tif]
