# Supplementary material for: ISWI and CHD Chromatin Remodelers Bind Promoters but Act in Gene Bodies
Source: PLoS Genet. 2013 Feb 28;9(2):e1003317. doi: 10.1371/journal.pgen.1003317 (PMC3585014; doi:10.1371/journal.pgen.1003317)
Supplement: Table S1 — Yeast strains used in this study. All strains are derived from W1588-4C, which is isogenic to W303-1A except that a weak RAD5 mutation is repaired [74]. (DOC) [file pgen.1003317.s007.doc]

| **Strain** | **Genotype** | **Source** |
| --- | --- | --- |
| YTT966 | *MAT***a** *ade2-1 can1-100 his3-11,15 leu2-3,112 trp1-1 ura3-1 RAD5+ ISW2-3FLAG-KanMX* | TT |
| YTT1448 | *MAT***a** *ade2-1 can1-100 his3-11,15 leu2-3,112 trp1-1 ura3-1 RAD5+ ISW1-3FLAG-KanMX* | TT |
| YTT1726 | *MAT***a** *ade2-1 can1-100 his3-11,15 leu2-3,112 trp1-1 ura3-1 RAD5+ CHD1-3FLAG-KanMX* | TT |
| YTT1996 | *MAT***a** *ade2-1 can1-100 his3-11,15 leu2-3,112 trp1-1 ura3-1 RAD5+ isw2-K215R-3FLAG-KanMX* | TT |
| YTT2266 | *MAT***a** *ade2-1 can1-100 his3-11,15 leu2-3,112 trp1-1 ura3-1 RAD5+ isw1-K227R-3FLAG-KanMX* | TT |
| GZY1 | *MAT***a** *ade2-1 can1-100 his3-11,15 leu2-3,112 trp1-1 ura3-1 RAD5+ chd1-K407R-3FLAG-KanMX* | GEZ |

**Table S1. Yeast strains used in this study**

All strains are derived from W1588-4C, which is isogenic to W303-1A except that a weak *RAD5* mutation is repaired.
