# Supplementary material for: ISWI and CHD Chromatin Remodelers Bind Promoters but Act in Gene Bodies
Source: PLoS Genet. 2013 Feb 28;9(2):e1003317. doi: 10.1371/journal.pgen.1003317 (PMC3585014; doi:10.1371/journal.pgen.1003317)
Supplement: Table S2 — Numbers of mapped pairs and supernucleosomal/nucleosomal AUC ratios for sequenced samples. ChIP/input is the ChIP supernucleosomal/nucleosomal AUC ratio divided by the input supernucleosomal/nucleosomal AUC ratio. (DOC) [file pgen.1003317.s008.doc]

| **Sample** | **Mapped pairs** | **Supernucleosomal/**  **nucleosomal** | **ChIP/input** |
| --- | --- | --- | --- |
| Isw1 WT ChIP, 2.5' MNase | 10,813,831 | 2.13 | 3.87 |
| Isw1 WT input, 2.5' MNase | 23,838,438 | 0.55 |  |
| Isw1 WT ChIP, 10' MNase | 11,703,996 | 0.43 | 2.26 |
| Isw1 WT input, 10' MNase | 27,265,131 | 0.19 |  |
| Isw1 K227R ChIP, 2.5' MNase | 69,191,301 | 1.31 | 3.36 |
| Isw1 K227R input, 2.5' MNase | 25,925,172 | 0.39 |  |
| Isw2 WT ChIP, 2.5' MNase | 70,853,363 | 1.32 | 2.16 |
| Isw2 WT input, 2.5' MNase | 11,195,170 | 0.61 |  |
| Isw2 WT ChIP, 10' MNase | 16,246,585 | 0.49 | 2.56 |
| Isw2 WT input, 10' MNase | 29,232,498 | 0.19 |  |
| Isw2 K215R ChIP, 2.5' MNase | 42,821,618 | 1.23 | 2.73 |
| Isw2 K215R input, 2.5' MNase | 9,547,428 | 0.45 |  |
| Chd1 WT ChIP, 2.5' MNase | 33,514,384 | 2.09 | 1.74 |
| Chd1 WT input, 2.5' MNase | 75,234,013 | 1.20 |  |
| Chd1 WT ChIP, 10' MNase | 26,500,798 | 0.58 | 2.76 |
| Chd1 WT input, 10' MNase | 13,765,555 | 0.21 |  |
| Chd1 K407R ChIP, 2.5' MNase | 62,993,924 | 3.32 | 2.79 |
| Chd1 K407R input, 2.5' MNase | 72,600,087 | 1.19 |  |

**Table S2. Numbers of mapped pairs and supernucleosomal/nucleosomal AUC ratios for sequenced samples**

ChIP/input is the ChIP supernucleosomal/nucleosomal AUC ratio divided by the input supernucleosomal/nucleosomal AUC ratio.
